# Fucoxanthin for Topical Administration, a Phototoxic vs. Photoprotective Potential in a Tiered Strategy Assessed by In Vitro Methods

**DOI:** 10.3390/antiox9040328

**Published:** 2020-04-17

**Authors:** Renata Spagolla Napoleão Tavares, Camila Martins Kawakami, Karina de Castro Pereira, Gabriela Timotheo do Amaral, Carolina Gomes Benevenuto, Silvya Stuchi Maria-Engler, Pio Colepicolo, Hosana Maria Debonsi, Lorena Rigo Gaspar

**Affiliations:** 1 School of Pharmaceutical Sciences of Ribeirão Preto, University of São Paulo, Ribeirão Preto, SP 14040-903 São Paulo, Brazil; renatasnt@usp.br (R.S.N.T.); hosana@fcfrp.usp.br (H.M.D.); 2 Clinical and Toxicological Analyses Department, School of Pharmaceutical Sciences, University of São Paulo, SP 05508-000 São Paulo, Brazil; 3 Institute of Chemistry, University of São Paulo, SP 05508-000 São Paulo, Brazil

**Keywords:** antioxidant, fucoxanthin, phototoxicity, photostability, reconstructed human skin

## Abstract

Fucoxanthin possesses a well-described antioxidant activity that might be useful for human skin photoprotection. However, there is a lack of scientific information regarding its properties when applied onto human skin. Thus, the objective of the present study was to assess the photoprotective and phototoxicity potential of fucoxanthin based on its ultraviolet (UVB 280–320 nm; UVA 320–400 nm) and visible (VIS 400–700 nm) absorption, photostability, phototoxicity in 3T3 mouse fibroblast culture vs. full-thickness reconstructed human skin (RHS), and its ability to inhibit reactive oxygen species formation that is induced by UVA on HaCaT keratinocytes. Later, we evaluated the antioxidant properties of the sunscreen formulation plus 0.5% fucoxanthin onto RHS to confirm its bioavailability and antioxidant potential through the skin layers. The compound was isolated from the alga *Desmarestia anceps*. Fucoxanthin, despite presenting chemical photo-instability (dose 6 J/cm^2^: 35% UVA and 21% VIS absorbance reduction), showed acceptable photodegradation (dose 27.5 J/cm^2^: 5.8% UVB and 12.5% UVA absorbance reduction) when it was added to a sunscreen at 0.5% (*w*/*v*). In addition, it increased by 72% of the total sunscreen UV absorption spectra, presenting UV-booster properties. Fucoxanthin presented phototoxic potential in 3T3 fibroblasts (mean photo effect 0.917), but it was non-phototoxic in the RHS model due to barrier function that was provided by the stratum corneum. In addition, it showed a significant inhibition of ROS formation at 0.01% (*p* < 0.001), in HaCat, and in a sunscreen at 0.5% (*w*/*v*) (*p* < 0.001), in RHS. In conclusion, in vitro results showed fucoxanthin protective potential to the skin that might contribute to improving the photoprotective potential of sunscreens in vivo.

## 1. Introduction

Concerning ultraviolet (UV) damage, at the beginning of this century, not many compounds were available for the protection against UVA (320–400 nm) radiation and, in response to the growing concern regarding the additional damage that is caused by this radiation, various UVA filters are now available for formulations worldwide. However, the number of UVA filters allowed in the USA is quite limited [1,2]. Another perspective is related to the fact that few compounds offer UVA I (340–400 nm) and visible (VIS 400–700 nm) light protection, with increasing evidence of the harmful effects of VIS light on the skin. Liebel and co-workers [3] showed that VIS light induced the generation of high levels of free radicals in humans and of proinflammatory cytokines in vitro.

Even conventional sunscreens containing UVA and UVB filters, active ingredients that absorb or reflect UV, do not entirely block the UV radiation that reaches the skin [4]; additionally, they can undergo photodegradation and produce reactive oxygen species (ROS) with a phototoxic potential [5]. Besides that, some UV filters have controversial data regarding their skin permeation, estrogen-like effects, and induction of cutaneous sensitization and photosensitization [6,7]. They might have harmful impact not only on the human skin, but also on marine life and coral reefs, other aquatic ecosystems, like phytoplankton, marine diatom, and alga growth [8,9]. In 2018, the governor of Hawaii banned the in-state sale of sunscreens containing either oxybenzone or octinoxate, suspected to harm coral reefs [10]. After Hawaii, Florida and Key West followed this trend. This movement has stimulated the urgent research on alternatives and possibly eco-friendly photoprotective compounds [10]. 

Following this trend, natural and biocompatible UV filters have led to research on carotenoids that could be interesting in the development of new UV filters or UV boosters to increase the protection or performance of sunscreens [11]. Booster is a term that is currently used in the cosmetic field to define substances that, in small amounts, are capable of increasing the effectiveness of some other products [11] (i.e., increasing the effects of UV filters protecting the skin from sunlight-induced ROS production).

Fucoxanthin absorbs from 320 to 500 nm (UVA I to VIS, 448 nm max) and its action might avoid UVA-induced photoaging and protect from VIS- induced ROS production [2,12]. Since fucoxanthin contains an alene bond, a conjugated carbonyl group, one 5,6-monoepoxide, and an acetyl group, there is a biological potential that is associated with this structure of the molecule when orally administered [13]. It has been reported that this carotenoid shows intense antioxidant activity, as well as anti-inflammatory, anti-obesity, anti-diabetic, anti-tumor, antihypertensive, and anticancer properties [13,14]. Many authors have suggested the photoprotective effect of fucoxanthin on the skin, including protection against UVB-induced damage when 0.001% of fucoxanthin is applied to hairless mice [15]. However, its topical properties and safety for the human skin are still unknown. 

Alternative in vitro methods validated for preclinical trials are being used to predict the safety and efficacy of unknown natural compounds instead of using animal models to evaluate the potential risk of a test chemical. The use of skin models is physiologically relevant in drug development, since it provides better prediction of human skin safety [16], besides ethical and economic concerns. Thus, for phototoxicity prediction, the recommended in vitro tiered strategy, including monolayer fibroblast 3T3 Neutral Red Uptake Phototoxicity (3T3 NRU PT) and reconstructed human epidermis (RHE), allows for the identification of phototoxic potential without animal testing [5,17,18]. Nevertheless, RHE does not present the dermal component, which is essential for many epidermal characteristics and proper skin functionalities, including the improvement of barrier function [19]. In our study, we replaced RHE with reconstructed human skin (RHS) to confirm the ability of the latter to detect the phototoxicity potential. Besides the evaluation of fucoxanthin photosafety, we evaluated the photoprotective potential of this molecule by its photostability under different irradiation exposure and when used in a sunscreen formulation. Additionally, we evaluated the maximal antioxidant potential of this molecule onto immortalized human keratinocytes (HaCat) cells, determining the ideal concentration range that is to be used for this effect. Later, we evaluated the antioxidant properties of the sunscreen formulation plus 0.5% fucoxanthin onto RHS to confirm its bioavailability and antioxidant potential through the skin layers. 

Thus, the objective of the present study was to assess the photoprotective and phototoxicity potential of fucoxanthin based on its UV/VIS absorption, photostability, phototoxicity in 3T3 mouse fibroblast culture vs. full-thickness RHS, and its ability to inhibit reactive oxygen species formation that is induced by UVA on HaCaT keratinocytes.

## 2. Materials and Methods

### 2.1. Alga Material

During the expedition of 2011 (January 4th, managed by the project PROANTAR (*Programa Antártico Brasileiro*), the researchers collected 69.00 grams of *D. anceps* (wet) at the Punta Plaza location—Antarctic Continent (Admiralty Bay 62°04′14.5″–62°10′03.5″ S and 58°20′15″–58°27′60″ W). The material was frozen until the time for transportation to our laboratories in Brazil, at the Laboratory of Organic Chemistry of the Marine Environment-Support Center for Research in Natural and Synthetic Products, School of Pharmaceutical Sciences of Ribeirão Preto (LQOAM-NPPNS, FCFRP-USP). The investigators preserved a sample of the material that was collected in a solution of formaldehyde with 4% seawater for morphological studies and the preparation of vouchers. They identified the macroalgae according to the standard taxonomic methodology in the Phycology Session and deposited exsiccates in the Phycological Herbarium of the Botanical Institute of São Paulo (SP), Brazil.

### 2.2. Extraction and Fractionation

We used a mass of 69.00 g (wet weight) to obtain the extract. The material was freshly thawed and washed with distilled water under a vacuum filter. Subsequently, it was fragmented and then extracted with the organic solvent dichloromethane (CH_2_Cl_2_): methanol (MeOH) (2:1) for 30 min. under stirring, in a thermal blanket with controlled temperature (not exceeding 30 °C). The material was filtered and extracted two more times while using ultrasound equipment for 15 minutes in the third procedure. We concentrated the organic *D. anceps* extract in a rotary evaporator under low pressure (Büchi R-300, Buchi Labotechnik, Flawil, Switzerland) and subjected it to a classical chromatographic column—30 cm with stationary phase Silica Gel 40–63 μm/ASTM Macherey-Nagel (Merck, Darmstadt, Germany), with a polarity gradient using n-hexane, ethyl acetate (EtOAc), and methanol (MeOH) (JT Baker, Port of Spain, Trinidad Y Tobago) to fractionate the extract.

### 2.3. Carotenoid Isolation

For the identification of fucoxanthin, the highest colored mass fractions were subjected to Electrospray Ionization Mass Spectrometry (ESI-MS) techniques (amaZon SL, Bruker, Bil-lerica, MA, USA). To isolate the carotenoid, we employed high-performance liquid chromatography (HPLC), analytical, and semi-preparative analyses while using two different types of equipment with three different columns. The first instrument and column used to yield the sub-purified fractions was a Shimadzu Chromatograph Model SCL-10AVP that was equipped with a Shimadzu diode array UV-VIS detector DAD (SPD-M10 AVP, Shimadzu, Kyoto, Japan), a computerized integration system Class-VP software 5.02 (Shimadzu, Kyoto, Japan) and the following chromatographic columns: analytical Supelco C-18 (25 cm × 4.4 mm, 5 μm) and semi-preparative LC-18 Supelco (25 cm × 10 mm, 5 μm). The second analytical column was Polar RP column (100 mm × 3 mm, 5 µm) that was used to define a method to purify the carotenoid (from chlorophyll “a” as a contaminant). The second instrument was Shimadzu chromatograph (Shimadzu, Kyoto, Japan), Prominence model, CBM-20th controller, SPD-20th detector UV/VIS, with two pumps (LC-6AD), an FCR-10th automatic collector DGU-20A5 degasser and LC-Solution Single Software, a semi-preparative column Synergi Polar-RP (250 mm × 10 mm, 4 mm), and a semi-preparative Polar-RP guard column (10 × 10 mm, 4 mm), both being from Phenomenex^®^ (Torrance, CA, USA).

### 2.4. Stability

#### 2.4.1. UV Absorption

Solutions of 100 μg/mL of extract (the ideal concentration for maximal absorbance around 1 AU), fractions, and fucoxanthin alone in isopropanol were analyzed with a spectrophotometer in the 280 to 700 nm range for the determination of the UV absorption spectra. 

#### 2.4.2. Photostability Studies

For the determination of photostability, we studied the crude *D. anceps* extract, the fraction Fr15 containing fucoxanthin, and fucoxanthin that were isolated from this fraction in an isopropanol solution at 100 μg/mL or dissolved in a sunscreen formulation at 0.5% (*w*/*v*). For the photostability studies in an organic solution, 1 mL of each solution sample was added to glass beakers and then subjected to solvent evaporation until a dried film was obtained. The samples were then submitted or not to UV radiation of 7 mW/cm^2^ emitted from a Philips UVA lamp Actinic BL/10 (Eindhoven, Netherlands) measured with a Dr. Hönle radiometer (Planegg, Germany) equipped with a UVA sensor [20,21,22,23]. For the *D. anceps* extract and Fr15, we applied a cumulative dose of 27.5 J/cm^2^. That dose is recommended as being similar to 66 min. of exposure to sunlight at midday (6.94 mW/cm^2^) on a typical September sunny day in the Ribeirão Preto, Brazil—latitude 21°10′39″ south and longitude 47°48′37″ west [4,23]. We applied a cumulative dose of 6 J/cm^2^ to determine the photostability of fucoxanthin in isopropanol (the dose recommended for phototoxicity studies and similar to 14 min. of exposure to sunlight at midday in the Ribeirão Preto region). Each beaker subjected to irradiation had a negative control that was sheltered from light. After irradiation, the dried film was resuspended in 1 mL of solvent, and the absorption spectrum of the solutions in the 280 to 700 nm range was analyzed. For the photostability study of fucoxanthin in a sunscreen formulation, we prepared the formulation base with a self-emulsifying wax (cetearyl alcohol, cetearyl glucosyde) and a liquid polymer (hydroxyethyl acrylate, sodium acryloyldimethyltaurate copolymer, squalane, and polysorbate 60), and, in the presence of the following UV filters: 4% avobenzone, 6% octocrylene, 8% octyl methoxycinnamate, and 3% octyl triazone, representing formulation “F3” in the Freitas [22] et al. (2015) study. We proceeded as indicated in the cited study to perform the photostability study using a sunscreen, spreading the preparation onto an area of 10 cm^2^ (approximately 4 mg/cm^2^) of a glass plate and then left to dry for 15 min. before exposure to a UVA dose of 27.5 J/cm^2^. We used the area under the curve (AUC), which is the integral of the absorption spectrum of the samples in the UVB (280–320 nm), UVA (320–400 nm), and VIS (400–700 nm) ranges using the integration function of the MicroCal OriginPro Software (8 SRO, OriginLab Corporation, Northampton, MA, USA) to calculate the photostability [24]. We expressed the results as a percentage of the area of irradiated samples related to the area of non-irradiated samples.

### 2.5. Toxicity and Efficacy

#### 2.5.1. Phototoxicity Test in 3T3 Mouse Fibroblast (3T3 NRU PT)

In this test, fibroblasts of the Balb 3T3 clone A31 that were provided by *Banco de Células do Rio de Janeiro,* BCRJ code 0047 (Rio de Janeiro, Brazil) cultured on two 96 well microtiter plates were pre-incubated with eight different concentrations of the test chemical (6.8–100 μg/mL) for one hour. We exposed one plate to a UVA irradiation dose of 9 J/cm^2^ (SOL-500 sun simulator that was equipped with a metal halide lamp and H1 filter, Dr. Honle AG, Planegg, Germany), while another one was kept in the dark. The determination of cell viability comparing the plates determinates the substance cyto- and phototoxicity [5,25]. Based on the historical data that was produced in our laboratory, we defined this dose as a dose high enough to elicit a phototoxic response in positive controls and as a dose that did not produce interference higher than 20% in cell viability, as recommended by the Organisation for Economic Co-operation and Development (OECD) [26]. We measured UVA radiation with the same radiometer mentioned before. For concentration-response analysis, we employed the Phototox Version 2.0 software, obtained from *Zentralstelle zur Erfassung und Bewertung von Ersatz- und Ergänzungsmethoden zum Tierversuch* (ZeBeT, Berlin, Germany) that calculates the photoirritation factor (PIF) and mean photo effect (MPE). According to the OECD Test Guideline 432, a substance is predicted to be phototoxic if MPE is higher than 0.15 or the PIF is higher than 5. A test substance with an MPE > 0.1 and < 0.15 (PIF > 1 and <5) is predicted to be “probably phototoxic” [26]. The positive control was norfloxacin purchased from Sigma–Aldrich (St. Louis, MO, USA) [26].

#### 2.5.2. Reconstructed Human Skin Model (RHS) 

The ethics committees of Human Research Ethics Committee of Faculty of Pharmaceutical Sciences of Ribeirão Preto, São Paulo, Brazil approved the experimental procedures using primary human fibroblasts and keratinocytes from donated foreskins with informed consent from legal representatives and ethics approval conforming to the principles of the Declaration of Helsinki (CAAE n°: 55438216.0.0000.5403). We constructed the dermal equivalent with 3.6 × 10^5^ fibroblasts, 5% fetal bovine serum (Gibco, Life Technologies, Carlsbad, CA, USA), and rat-tail type I collagen (Corning, Tewksbury, MA, USA) enough to 3 mL per insert six-well-plate-size (Corning, Tewksbury, MA, USA). After 2 h, we added 2.9 × 10^6^ of the primary human keratinocytes on top of the dermis equivalent using 2 mL of keratinocyte medium. They were kept submerged for 24 h. Next, the culture was maintained at the air-liquid interface for 12 days to allow for keratinocytes differentiation [27]. 

#### 2.5.3. Phototoxicity Test in RHS

On the 10th day of tissue cultivation, the skin models were exposed to fucoxanthin solubilized in c12-c15 alkyl benzoate (0.5%, *v*/*v*), which is a vehicle that is commonly used to solubilize lipophilic chemicals in cosmetics [5]. Sterile filter discs 16 mm in diameter were soaked in 50 µL test chemicals (c12–c15 alkyl benzoate) and directly applied to the stratum corneum of the skin models. Ketoprofen at 3% in alkyl benzoate is phototoxic and it was used as a positive control. Twenty hours after application of the test substances, the skin models were rinsed with phosphate-buffered saline (PBS), dried with a sterile swab, and then transferred to fresh wells with the medium. The skin models were irradiated (sun simulator mentioned before) with 6 J/cm^2^, the dose that was recommended by Kandarova and Liebsch [28], as necessary to produce a phototoxic response in the positive controls without damage to the tissue and the one used to pre-validate the test in 1999 by European Centre for the Validation of Alternative Methods (ECVAM) [29]. We measured UVA radiation with the same radiometer mentioned before, while we kept the non-irradiated plates in a dark box.

#### 2.5.4. Viability Assay

RHS viability was measured by the end of the experiment of phototoxicity. It was determined by measuring the metabolic activity of the constructs after exposure and post-incubation while using a colorimetric test. The reduction of mitochondrial dehydrogenase activity was assessed via the decreased formazan production following incubation with MTT (3-(4,5-dimethylthiazol-2-yl)-2,5-diphenyltetrazolium bromide, Sigma-Aldrich, Cotia, Brazil). The formazan production was measured at 570 nm. We exposed other constructs in every batch to fucoxanthin, but not to MTT, to evaluate the ability of fucoxanthin to stain the constructs under test conditions. The formazan readings were corrected by the fucoxanthin-related optical densities (O.D.) and compared to those of negative control RHS [30]. The data are presented as relative viability according to Equation (1), where “*O.D.a*” is from tissues treated and “*O.D.b*” is the mean of untreated tissues.

(1) Relative viability(%)=100×O.D.bmean O.D.b

#### 2.5.5. HaCat Antioxidant Activity by Detection of Intracellular ROS Using DCFH_2_-DA

The keratinocytes HaCaT that were provided by *Banco de Células do Rio de Janeiro,* BCRJ code 0341 (Rio de Janeiro, Brazil) were seeded in two 96-well plates at a density of 1 × 10^5^ cells/well and then incubated at 37 °C in a 5% CO_2_ atmosphere for 24 h. Subsequently, cells were treated for one hour with fucoxanthin at 0.1, 1, 10, and 100 µg/mL (the same range of concentrations used for 3T3 NRU PT of 6.8–100 µg/mL). Next, we incubated the plates with the probe 2′,7′-dichlorodihydrofluorescein diacetate (DCFH_2_-DA) (10 µM) in the dark for 30 min. for permeation into the cell. After the period of incubation, we irradiated one plate with 4 J/cm^2^ of UVA radiation (sun simulator), while the other one we kept in dark box. Immediately after irradiation, fluorescence intensity was measured with a microplate reader (BioTek Synergy HT, Winooski, VT, USA) at an excitation wavelength of 485 nm and emission wavelength of 528 nm [31,32]. We expressed the results as percent fluorescence intensity when compared to the untreated control irradiated, which was considered to be 100%. Norfloxacin (100 µg/mL) and quercetin (10 µg/mL) were used as controls. All of the experiments were carried out in triplicate in three independent experiments. The experimental data was analyzed statistically by analysis of variance (ANOVA), a parametric test, followed by Tukey’s test.

#### 2.5.6. RHS Antioxidant Activity by Detection of Intracellular ROS Using DCFH_2_-DA

The RHS were incubated with the DCFH_2_-DA probe in PBS (50 µM) in the dark for 45 min. After PBS washing, the tissues were treated with sunscreen with fucoxanthin at 0.5% (as described in 2.3.2 item) and the control was the sunscreen formulation without it, for 1 h. Subsequently, they were subjected to a dose of 10 J/cm^2^ of UVA radiation, while control tissues were kept in the dark. Immediately after the irradiation period and PBS washing, the tissues were frozen in liquid nitrogen and 10 mm cryostat sections were made. The fluorescence intensity was measured while using a Ti-S inverted Microscope (Nikon Instruments Inc., Amsterdan Netherlands), 488 nm, at 100 ms exposition intensity. The images were quantified using Image J software [33,34]. The results were normalized to area/pixels and expressed in a percentage of fluorescence intensity in comparison to the irradiated and non-irradiated untreated controls.

## 3. Results

### 3.1. Extraction and Fractionation

We obtained the dry extract (1.29 g) from fresh wet alga material (69.00 g), with a yield of 2.31%. Next, 1.15 g of the extract was submitted to fractionation in a classical column, which resulted in 40 fractions. We selected fractions that yielded higher weight to have enough mass for all of the following tests; firstly, the selected fractions were screened in terms of their UV spectra.

### 3.2. UV Spectra

**Figure 1:**
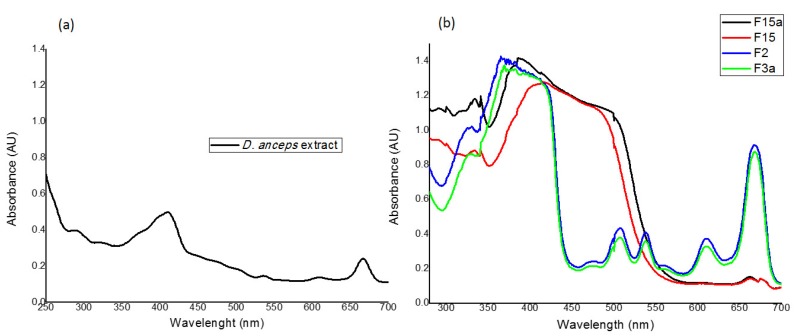
Absorption spectra of (**a**) The alga *D. anceps* crude extract; isopropanol solution, 100 μg/mL; (**b**) The fractions obtained from this crude extract presented different spectrum profiles; F = fraction, numbers ordered by elution (nonpolar to polar “a” indicates the second day of the extraction. Representative curves from triplicates.

*D. anceps* extract presented absorption in the region of interest for photoprotection (280–400 nm), especially in the UVA and in the visible range (Figure 1a). Concerning brown algae, the typical absorptions in the regions of 400 nm and 660 nm (areas of blue and red, respectively) are due to the presence of carotenoids and chlorophylls, especially “chlorophyll a”, which is common to all photosynthetic organisms having a maximum absorption at 420 and 660 nm, respectively. Chlorophyll type “a” is predominant in algae due to its central role in the conversion of photochemical energy, while the chlorophyll “c” efficiently participates in photosynthesis as an accessory pigment (similar to the role of chlorophyll “b” in plants or green algae) [14]. 

The spectral composition of light is crucial for photoprotection mechanisms and photosynthetic efficiency and, thus, for the pigment content of macroalgae as well. According to Kuczynska et al. [35], different structures of chlorophyll “c” are responsible for its intense absorption in the region of 530 nm; also, a peak in the 680 nm region (Figure 1a) could be related to “chlorophyll a”. Carotenoids, on the other hand, exhibit intense absorption between 400 and 500 nm, after fractionation (Figure 1b), F15, F15a, F2, and F3a showed high UVA I/VIS absorbance. 

### 3.3. Identification and Isolation of Fucoxanthin

The high-resolution molecular mass spectrum (electrospray ionization, ESI) corresponding to the molecular weight of fucoxanthin, 658.90 g/mol, was identified based on the fragment pattern at *m/z* 659.4241 and 681.3878 corresponding to [M + H]^+^ and [M + Na]^+^ in the fractions F15 and F15a. We subjected the isolated fucoxanthin to NMR spectroscopy for its structural determination while comparing it with the literature (Table 1). The complete assignments of the ^1^H and ^13^C NMR spectra of fucoxanthin revealed signals that were assignable to polyene containing acetyl, conjugated ketone, olefinic methyl, two quaternary germinal oxygen methyls, two quaternaries geminal dimethyl, and allene groups. The NMR data are in line with the findings of Xia and coworkers [14] and Mori and coworkers [36], which suggested that fucoxanthin isolated from the alga *D. anceps* is mainly present in the all-*trans* form. By their coloring feature, other pigments, such as any xanthophyll common to brown algae, such as *cis*-fucoxanthin, diadinoxanthin, diatoxanthine, and β-carotene, could be present in the alga extract in addition to fucoxanthin [12]. These carotenoids also have a maximum absorption peak around 448 nm, as is the case for chlorophylls (b), (c), and (a), which are the main chlorophyll pigments in algae [14]. For the isolation of fucoxanthin from other carotenoids and chlorophylls, we performed an analytical chromatographic analysis with an exploratory method to verify the presence of compounds that absorb near 450 nm of fractions F15/F15a (higher yield and absorbance at 448 nm).

We established the method after optimization of analytical chromatographic elution: acetonitrile and water permitted sub-fractionation. The peak with the 47 min. retention time eluted the substance of interest that was monitored by absorption (λ max ~ 450 nm) (Supplementary Figure S1); chlorophyll, (a; c) together with fucoxanthin. Since these algal pigments are complexed, there is greater difficulty in separating them with superior purity [14]. However, after we modified the stationary phase, the mobile phase, and optimized the method employed in analytical chromatographic elution, we could separate them with a rough interval of 3 min (Supplementary Figure S2 or S3). 

### 3.4. Photostability Studies

**Figure 2:**
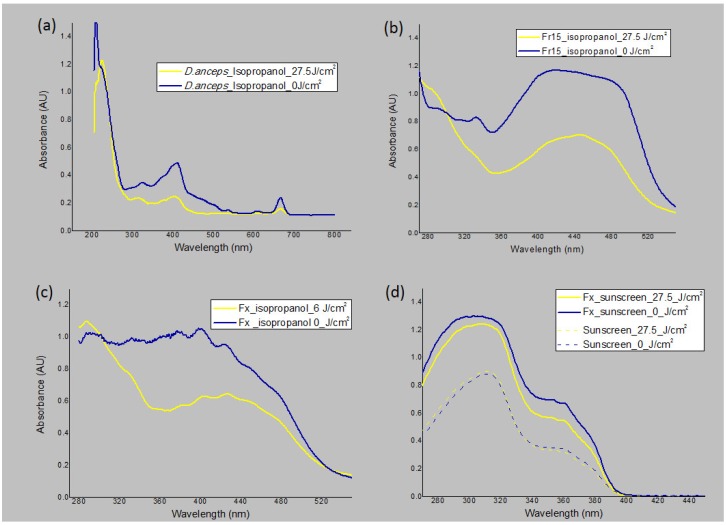
Photostability studies based on the electromagnetic spectrum of the samples irradiated (yellow line) or not (blue line). (**a**) *D. anceps* extract in solution irradiated or not with 27.5 J/cm^2^. (**b**) Fr15 fraction containing fucoxanthin in solution irradiated or not with 27.5 J/cm^2^. (**c**) Fucoxanthin (Fx) in solution irradiated or not with 6 J/cm^2^. (**d**) Fx in a sunscreen formulation vs. sunscreen alone (dashed line) irradiated or not with 27.5 J/cm^2^; *n* = 3, three independent experiments.

When irradiated with UVA at 27.5 J/cm^2^, the crude extract was considered to be photo unstable in the UV/VIS range (more than 20% reduction), since there was a 28.5%, 43.2%, and 33.7% decrease in UVB, UVA, and VIS absorption, respectively (Figure 2a, Table 2). Fraction F15 (fucoxanthin rich fraction) with maximal absorbance around 450 nm was considered to be photostable in UVB, with only a 4% decrease of absorbance; however, it was photo unstable in the UVA/VIS regions with 44% and 49% absorbance depletion, respectively (Figure 2b, Table 2). We then evaluated fucoxanthin that was isolated in isopropanol solution using the same UVA dose used to induce phototoxicity to RHS (6 J/cm^2^), in order to compare both of the experiments. With this lower dose, we observed no degradation in the UVB region, 35% in the UVA region, and 21% in the VIS region; the depletion of absorption of the isolated compound was not in the range considered to be photostable (Figure 2c) [2,4,37]. We repeated the photostability assay applying 0.5% (*w*/*v*) of pure fucoxanthin in a sunscreen formulation, formulation 3 (F3), based on the photostability studies of Freitas et al. [2]. The final sunscreen formulation was yellow-colored, but it was not able to stain the skin. When we added this marine carotenoid to a sunscreen formulation and irradiated with 27.5 J/cm^2^, it was considered photostable (5.8% reduction in UVB and 16.5% reduction in UVA). However, since the sunscreen alone only provoked a reduction in the UVA absorption by 4%, the effect of fucoxanthin on UVA region was considered to be the difference of 12.5%. Additionally, the sunscreen with fucoxanthin increased the general UV absorbance by 72% compared with the sunscreen alone, proving to act as a UV-booster by light-absorbing effect, Figure 2d.

### 3.5. Phototoxicity in the Monolayer and RHS Assays

The prediction model classified the positive control (norfloxacin) as phototoxic and within the MPE range that was recommended by the OECD Test Guideline 432 (0.340 to 0.900) [26]. The crude extract was considered cytotoxic (IC50-UV 2.7 µg/mL), and its phototoxicity could not be assessed since it compromised all the cells when evaluated in the concentration range of 6.4 to 100 g/mL (Table 3). Fraction F15a containing fucoxanthin was considered less cytotoxic than the crude extract (IC_50_ − UV 26.12 and 25.22 µg/mL) but was deemed to be phototoxic (MPE: 0.343 and 0.478). Fucoxanthin only showed phototoxic potential (MPE: 0.920 and 0.915) and no cytotoxic potential (IC_50_ not determined in the range of 6.4 to 100 µg/mL) to fibroblasts 3T3 (Table 3).

Regarding the phototoxic potential of fucoxanthin, its molecular weight is high, 658.90 g/mol, which indicated that this substance would have a reduced bioavailability through the stratum corneum and stratified keratinocytes layers of the skin, which could lead to the absence of phototoxicity in vivo when topically applied. To confirm this hypothesis, we evaluated fucoxanthin for the phototoxicity potential in the RHS model, at 0.5% (*w*/*v*), which is within the concentration range that is usually employed for antioxidants in a cosmetic formulation (0.01%–1%).

RHE and RHS, due to the presence of a stratum corneum, appear to be capable of detecting known human dermal phototoxicants. Consequently, under adequate test conditions, a negative result in a 3D skin model indicates that the acute photoirritation potential of the formulation can be regarded as low. In this case, negative test results do not generally preclude further clinical photosafety assessment while using the to-be-marketed formulation [29].

**Figure 3:**
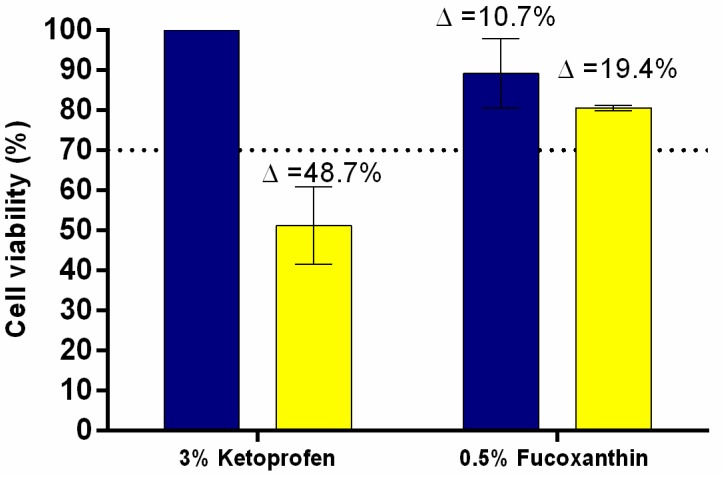
Phototoxicity assay using in-house Reconstructed human skin (RHS)—MTT assay, the positive control 3% ketoprofen was phototoxic (∆ > cut-off 30%) and 0.5% fucoxanthin was non-phototoxic (cell viability ~ 99%) when irradiated (yellow bar) or not (blue bar), mean ± SD, *n* = 2 in two independent experiments.

The positive phototoxic control 3% ketoprofen was correctly classified, since it showed a reduction of approximately 41% in cell viability when compared to the non-irradiated tissue, which is higher than the cut off value of 30% reduction that was determined for the assay (Figure 3). In contrast, fucoxanthin reduced less than 30% in cell viability and showed 8.7% difference between the irradiated and non-irradiated RHS models, proving to be non-phototoxic at this concentration onto RHS.

### 3.6. HaCat Antioxidant Activity by Detection of Intracellular ROS using DCFH_2_-DA

**Figure 4:**
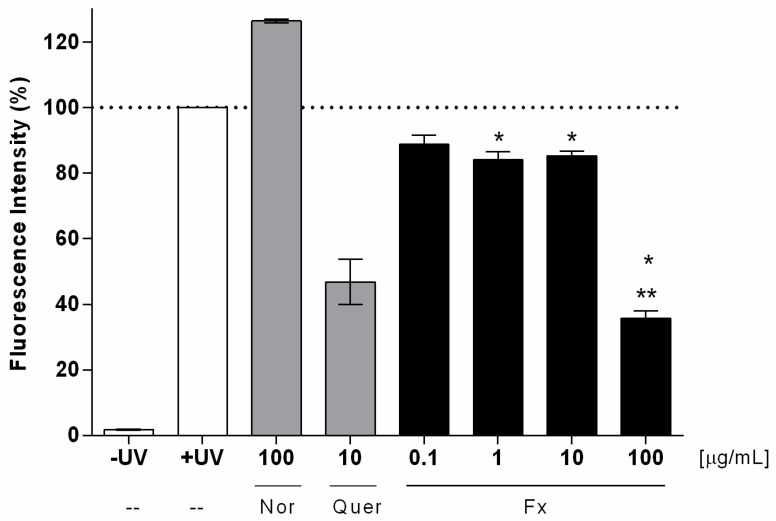
Intracellular Reactive Oxygen Species generation in HaCat after UVA irradiation (4 J/cm^2^) using a fluorescent probe DCFH2-DA; untreated irradiated (+UV) and non-irradiated (−UV); norfloxacin—Nor (control); quercetin—Quer (control); fucoxanthin—Fx. Where “*” means significantly different from untreated control irradiated (+UV) (*p* < 0.001) and “**” significantly different from fucoxanthin at 0.1, 1, and 10 µg/mL (*p* < 0.001). Mean ± SD, *n* = 3 in three independent experiments.

After the non-phototoxic response that was observed in the RHS, the protective effect of fucoxanthin was evaluated by the detection of intracellular ROS immediately after UVA radiation using a probe 2′,7′-DCFH_2_-DA in keratinocytes HaCat. The dose of 4 J/cm^2^ used was defined by many authors, including Chignell and Sik [38], after no significant difference in viability was observed between control cells and cells exposed to this dose higher than 20%, while an increase in the DCF photochemical reactions (ROS formation) was observed [38]. Cellular esterases hydrolyze the probe that is cell-permeable to the non-fluorescent DCFH derivative [39]. In the presence of hydrogen peroxide, hydroxyl radicals, carbonate, and nitrite, DCFH is oxidized to the highly fluorescent DCF, which can be monitored by several fluorescence-based techniques derivative [39,40]. The results demonstrated that UVA radiation-induced ROS generation in the keratinocyte cell line (100%) when compared to the untreated control non-irradiated. The positive control norfloxacin (100 µg/mL) induced an increase of ROS production of around 25% in comparison to the untreated irradiated control (Figure 4). On the other hand, the antioxidant quercetin (10 µg/mL) induced a reduction of ROS generation of around 46% after UVA irradiation (Figure 4). The treatment with fucoxanthin at 1, 10, and 100 µg/mL induced a significant reduction of ROS production of about 17%, 15%, and 65%, respectively, when compared to the untreated irradiated control (*p* < 0.001) (Figure 4). However, this effect was not observed for fucoxanthin 0.1 µg/mL, a dose that provided a reduction of 12%, which was not considered statistically significant when compared to the control (*p* > 0.05). Furthermore, treatment with fucoxanthin at 100 µg/mL induced the maximal reduction of ROS production, which was statistically different from the other studied concentrations (0.1, 1, and 10 µg/mL) (*p* < 0.001). 

### 3.7. RHS Antioxidant Activity by Detection of Intracellular ROS Using DCFH_2_-DA 

**Figure 5:**
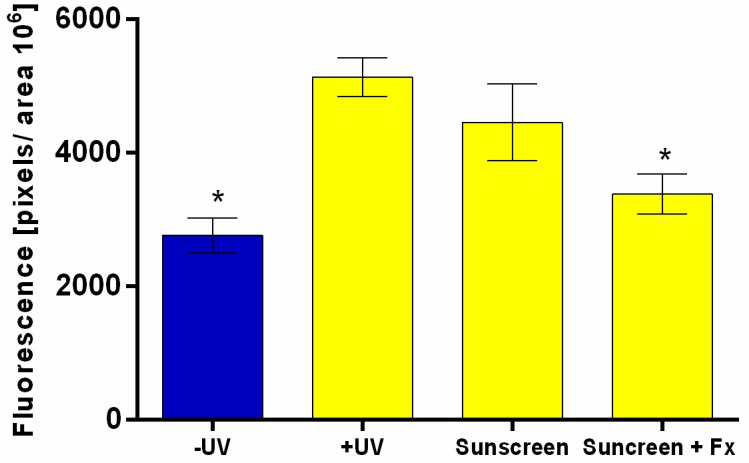
Reconstructed human skin after UVA irradiation (10 J/cm^2^) using a fluorescent probe DCFH_2_-DA. Reactive Oxygen Species generation quantified by the fluorescent intensity in pixels/area. Untreated (blue bar non-irradiated and yellow bars irradiated); sunscreen and sunscreen plus 0.5% fucoxanthin (Fx). Where “*” means significantly different from untreated irradiated (+UV) and from sunscreen treated models (*p* < 0.001). Mean ± SD, *n* = 3 in three independent experiments.

**Figure 6:**
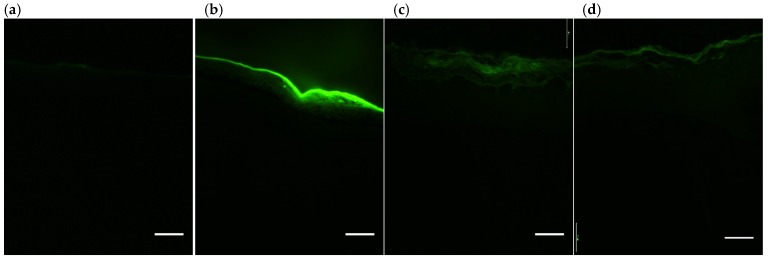
Representative reconstructed human skin (RHS) sessions (10 µm) exposed to a fluorescent probe DCFH_2_-DA and submitted or not to UVA irradiation (10 J/cm^2^)—(**a**) untreated non-irradiated; (**b**) untreated irradiated; (**c**) sunscreen irradiated; and, (**d**) sunscreen plus 0.5% fucoxanthin irradiated. Green fluorescent intensity represents the Reactive Oxygen Species (ROS) generation in the tissues. Scale bars = 100 µm, *n* = 3; three independent experiments.

Following the same mechanism of the probe DCFH_2_-DA, but this time with a sunscreen containing or not 0.5% of fucoxanthin applied in RHS tissues [33,34], the results demonstrated that the UVA radiation increased ROS generation in the untreated RHS control irradiated when compared to the untreated and not irradiated control (*p* > 0.05), (Figure 5 and Figure 6a,b). On the other hand, the sunscreen containing fucoxanthin 0.5% (*w*/*v*) induced a significant reduction of ROS generation after UVA irradiation when compared to the untreated control irradiated (+UV) and when compared to the sunscreen without fucoxanthin (Figure 5 and Figure 6a,d). RHS models only treated with sunscreen induced similar ROS production than untreated control irradiated (+UV) (*p* > 0.05) (Figure 5 and Figure 6c,d). 

## 4. Discussion

The marine carotenoid fucoxanthin that was isolated in this study from the alga *D. anceps* was the main carotenoid present in this brown alga [41]. It absorbs in the UV/VIS region, which is interesting for photoprotection and presents promising antioxidant properties that are observed in mice reported in the literature [12]. In the present study, fucoxanthin was, for the first time, evaluated in vitro by the toxicological and photoprotective perspective of its use in the human skin using 3D RHS. 

Fucoxanthin showed photoinstability when being evaluated in an organic solvent. However, UV filters or boosters should be stable under UV exposure, since their exposure to sunlight might lead to photodegradation reactions that can compromise their physical properties and lead to the formation of undesirable photoproducts [42]. Zhang and Tang [43] discussed that the instability of carotenes is related to the poly chain conjugate and its susceptibility to oxidation, isomerization by heat, light, and chemical interactions. After analyzing the chemical UVA/VIS photoinstability of fucoxanthin, with degradation being over the acceptable range of 20% after a 6 J/cm^2^ UVA dose, we added fucoxanthin at 0.5% to a sunscreen formulation to assess photostability at a 27.5 J/cm^2^ UVA dose. In this study, the sunscreen formulation that contains a combination of two UV filters considered to be photo unstable (avobenzone and ethylhexyl methoxycinnamate) was studied in order to assess the fucoxanthin capabilities of alteration in the photostability of this photo unstable combination. However, the sunscreen plus fucoxanthin showed acceptable photodegradation (Figure 2d). Surprisingly, besides that, we observed that the sunscreen plus fucoxanthin enhanced UVA and UVB absorption by 72%, showing in vitro booster properties of fucoxanthin that should be further investigated.

According to ICH [44], the intrinsic photostability of a substance or product shall be demonstrated in such a way that exposure to light does not result in unacceptable changes [44].

Addressing the tiered strategy to assess acute phototoxicity according to ICH recommendations [18], 3T3 NRU-PT is the first step of the biological assays and it is considered to be a standalone test for negative results due to its high sensitivity (100%) for the identification of absence of phototoxic potential [45]. When a positive result is obtained, i.e. fucoxanthin showed a phototoxic potential (MPE: 0.917), a follow-up testing should be performed to obtain data with models that better reflect the human situation, such as 3D skin models [45], since the 3T3 NRU-PT test is overestimated and it can produce false-positive results due to the lack of stratum corneum [28]. Firstly, our 3D full-thickness skin model proved to be able to detect the phototoxic potential of positive control, reducing the viability by more than 30% [28]. Secondly, fucoxanthin at 0.5% did not present phototoxicity (Figure 4). Therefore, the combined phototoxicity assays (monolayer and RHS) suggest that fucoxanthin would not be phototoxic to the human skin at 0.5% due to the reduced bioavailability through the stratum corneum and in the stratified epidermis. It is important to mention that skin models are more permeable than human skin [46], which means that they hardly produce false-negative results, which is very important when predicting the toxic potential of unknown substances. In addition, the viable epidermis is made of keratinocytes, which are less sensitive than fibroblasts to xenobiotics and UV radiation. 

In the efficacy assay on monolayers, fucoxanthin not only did not harm the HaCat keratinocytes, but also protected the cells from UVA-induced ROS formation in a concentration-dependent manner (maximal effect at 0.01%) (Figure 4). The reduction in ROS production was probably due to its antioxidant properties under anoxic conditions, well described by other studies using in chemico (DPPH; ABTS) [14] and in vitro methods (HepG2, HaCat, PC12) [47]. While the other carotenoids have virtually no scavenging ability against ROS, fucoxanthin donates electrons as a part of its mechanism of capturing free radicals [12]. Furthermore, we also observed a significant reduction in the ROS production in RHS (complex 3D tissue) treated and irradiated with sunscreen plus fucoxanthin that we did not observe in the RHS that was treated with sunscreen alone (Figure 5 and Figure 6). This suggests that fucoxanthin can also protect viable epidermis against UVA-induced ROS production, which is in agreement with the fucoxanthin protection observed in HaCat keratinocytes. These results could be considered to be very positive for the risk-benefit of its dermatological use, since fucoxanthin that reaches viable epidermis protects against UVA-induced ROS production (Figure 6d). 

Our study corroborates those reported before [48], which employed the HaCat cell line and hairless mice to study the UVB protective effects of commercial all-trans-fucoxanthin (Sigma). They observed that fucoxanthin has anti-inflammatory activity by downregulating Cyclooxygenase-2 (COX-2) expression after UVB irradiation (mice) and a photoprotective effect against oxidative stress that is caused by UVB exposure via an increase of nuclear factor E2-related factor 2 (Nrf2) expression. Thus, although Rodríguez-Luna et al. [48] obtained valuable information from hairless mice, it is well known that human adverse events cannot always be detected in animals, due to inherent genetic and physiological differences between species and that skin model constructed with human cells and physiologic relevant microarchitecture contributes to the better prediction of human effects. In the previous study of our group, commercial fucoxanthin at the same concentration, presented an absence of irritation, absence of morphological changes in the histology, and no significant dysregulation on homeostasis, metabolism, and in the inflammatory genes studied after following the OECD protocol for skin irritation [49]. 

Finally, it is important to mention that another well-known molecule, β-Carotene, a vitamin A precursor, is a popular “secondary” UV filter (characterized by a sun protection factor lower than 2), has a controversial use in the literature, regarding its antioxidant/prooxidant effect, depending on its concentration and O_2_ tension [2,22,50,51,52]. However, β-Carotene is able to protect the collagen structure from infrared light in the skin [53] and it is used in cosmetic formulations for aged skin and actinic keratosis [51], which, for instance, could also suggest some skin benefits from the topical use of fucoxanthin when tested in vivo, due to the similarities of their molecular structures. In addition, fucoxanthin when orally administered in mice is reported to have low accumulation in the skin [54], which underscores the interest of topical use of fucoxanthin and such confirmation of its safety and effectiveness suggested by the present study. 

## 5. Conclusions

In conclusion, we developed an effective extraction and purification of all-*trans*-fucoxanthin from the brown alga *D. anceps.* Although fucoxanthin presented chemical UV photo-instability, it increased by 72% of the total sunscreen UV absorption spectra (UVA and UVB) when added to sunscreen at 0.5%, presenting UV-booster properties with an acceptable photostability. The beneficial use of topical fucoxanthin should be further investigated in vivo in the concentration range of 0.01–0.5% (*w*/*v*). As, in this range, we observed an absence of phototoxicity in the RHS model and protective potential against UVA-induced ROS formation both in HaCat monolayers and on RHS models, which might contribute to improving the photoprotective potential of sunscreens.

## Figures and Tables

**Table 1:** Nuclear magnetic resonance of hydrogen and carbon (NMR ^1^H and ^13^C) of fucoxanthin (500 and 125 MHz, CDCl_3_) in comparison with literature data (400 and 67.5 MHz, CDCl_3_).

Position		^1^H (δ; mult; *J*-Hz)		^13^C (*δ*)	
		Literature *	Fucoxanthin	Literature *	Fucoxanthin
1				35.6	35.5
2	ax eq	1.36 dd (8.7; 14.2)	1.36 m	46.9	46.9
3		3.80 m	3.84 m	64.2	64.0
4	ax eq	1.77 dd (8.7; 14.2) 2.29 dd (2.9; 17.8)	1.77 dd (9.1; 13.9) 2.30 t (13.5)	41.5	41.3
5				66.0	67.0
6				66.9	66.8
7		3.64 d (20.4) 2.59 d (20.4)	3.65 d (18.4) 2.60 d (18.4)	40.6	40.0
8				197.7	197.7
9				134.3	134.4
10		7.14 d (12.8)	7.15 d (10.4)	139.0	139.0
11		6.58 m	6.57 m	123.2	
12		6.66 t (12.8)	6.66 t (11.3)	144.9	144.9
13				135.3	
14		6.40 d (11.6)	6.41 d (11.7)	136.6	136.4
15		6.67 m	6.66 m	129.3	
16	−Me	1.02 s	1.03 s	24.9	24.4
17	−Me	0.95 s	0.96 s	28.0	28.0
18		1.21 s	1.21 s	21.0	21.0
19		1.93 s	1.93 s	11.7	11.7
20		1.98 s	1.99 s	12.8	12.7
C-3’OAc	−Me	2.03 s	2.03 s	21.3	21.6
1’				35.0	35.5
2’	ax eq	1.41 dd (10.4; 14.9) 2.00 dd (2.9; 14.9)	1.42 d (11.9) 1.99 m	45.2	45.3
3’		5.37 tt (8.8; 12.0)	5.38 m	67.8	67.7
4’	ax eq	1.53 dd (10.4; 14.9) 2.29 dd (2.9; 17.8)	1,51 t (11.9) 2.30 m	45.1	45.1
5’				72.6	72.5
6’				117.3	117.3
7’				202.2	
8’		6.04 s	6.05 s	103.2	103.1
9’				132.4	132.3
10’		6.12 d (11.6)	6.13 d (11.1)	128.4	128.1
11’		6.71 t (12.0)	6.75 t (12.1)	125.5	
12’		6.34 d (11.6)	6.35 d (15.0)	137.0	136.4
13’				138.0	
14’		6.26 d (11.6)	6.27 d (11.5)	132.0	
15’		6.71 dd (12.0; 14.2)	6.75 t (13.7)	132.4	
16’	−Me	1.37	1.38 s	29.0	29.2
17’	−Me	1.065	1.07 s	31.9	32.0
18’		1.345	1.34 s	31.1	31.2
19’		1.805	1.81 s	13.9	13.9
20’		1.985	1.98 s	12.8	12.7
21				170.0	170.4

* Mori and coworkers, 2004 [36].

**Table 2:** Reduction of absorbance after irradiation with different doses of UVA light expressed in percentage. Calculation considering the Area under the Curve (AUC) between samples irradiated or not in triplicates.

Sample	Irradiation Dose (J/cm^2^)	Mean of the Reduction of Absorbance after Irradiation (%)		
		UVB	UVA	VIS
Crude Extract	27.5	28.5	43.2	33.7
Fraction F15 *	27.5	4.0	44.0	49.0
Fucoxanthin Isolated	6 **	0.0	35.0	21.0
Fucoxanthin in Sunscreen	27.5	5.8	16.5	NE

* Fucoxanthin rich fraction, ** dose defined as enough to induce phototoxic responses, NE: Non-evaluated (sunscreen with absence of absorbance in the VIS range).

**Table 3:** Data from the 3T3 fibroblasts phototoxicity assay of the promising alga fractions, pure fucoxanthin, and the positive control (norfloxacin).

Chemical	IC_50_ − UV	IC_50_ + UV	MPE	PIF	Result
Extract	2.76	3.05	−0.014	0.90	cytotoxic
Fraction F15a	26.12	4.45	0.343	7.08	photo/cytotoxic
	25.22	2.59	0.478	16.13	photo/cytotoxic
Fucoxanthin	-	2.77	0.920	48.21	phototoxic
	-	5.91	0.915	17.04	phototoxic
Positive Control (Norfloxacin)	-	2.487	0.615	43.75	phototoxic

*n* = 1 or 2 independent experiments, -: values not determined in the studied concentration range

## Supplementary Materials

The following are available online at https://www.mdpi.com/2076-3921/9/4/328/s1, Figure S1: Chromatogram of the elution semi-preparative scale—carotenoid isolation, Figure S2: Chromatogram of the semi-preparative scale subfraction, analyzed in 450 nm—absorption region of fucoxanthin at a higher intensity (Au 450), Retention Time (RT) = 5.75 min black line, 650 nm—region of the band’s absorption of chlorophyll “a” lower intensity. RT ~3 min, pink line, Figure S3: Chromatogram of the elution of the subfraction analytical scale, analyzed in (a) the 450 nm—absorption region of fucoxanthin at a higher intensity (3 Au), Retention time (RT) = 3.5 min; (b) 650 nm—the area of the chlorophyll absorption bands at a lower intensity (0.025 Au), RT ~6.5 min.
