# Supplementary material for: Fucoxanthin for Topical Administration, a Phototoxic vs. Photoprotective Potential in a Tiered Strategy Assessed by In Vitro Methods
Source: Antioxidants (Basel). 2020 Apr 17;9(4):328. doi: 10.3390/antiox9040328 (PMC7222355; doi:10.3390/antiox9040328)
Supplement: Supplementary file 1 [file antioxidants-09-00328-s001.pdf]

antioxidants

MDPI

Supplementary Figures

Article

Fucoxanthin for Topical Administration, a Phototoxic vs Photoprotective Potential in a Tiered Strategy Assessed by in vitro Methods

Renata Spagolla Napoleão Tavares [1], Camila Martins Kawakami [1], Karina de Castro Pereira [1], Gabriela Timotheo do Amaral [1], Carolina Gomes Benevenuto [1], Silvya Stuchi Maria-Engler [2], Pio Colepicolo [3], Hosana Maria Debonsi [1], and Lorena Rigo Gaspar [1,*]

[1] School of Pharmaceutical Sciences of Ribeirão Preto, University of São Paulo, SP 14040-903, Ribeirão Preto, São Paulo, Brazil; renatasnt@usp.br (R.S.N.T.); milakawakami@hotmail.com (C.M.K.); karina.pereira@usp.br (K.C.P.); gabriela.timotheo.amaral@usp.br (G.T.A.); carolgb@fcfrp.usp.br (C.G.B.); hosana@fcfrp.usp.br (H.M.D.)

[2] Clinical and Toxicological Analyses Department, School of Pharmaceutical Sciences, University of São Paulo, SP 05508-000, São Paulo, Brazil; silvya@usp.br

[3] Institute of Chemistry, University of São Paulo, SP 05508-000 São Paulo, Brazil; piocolep@iq.usp.br

* Correspondence: lorena@fcfrp.usp.br; Tel.: +55 16 33154315

Received: 18 February 2020; Accepted: 13 March 2020; Published: date

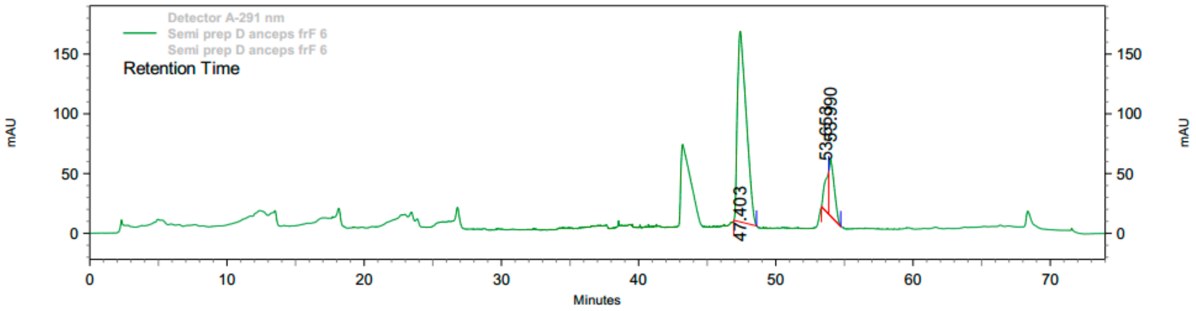

Figure S1. Chromatogram of the elution semi-preparative scale—carotenoid isolation.

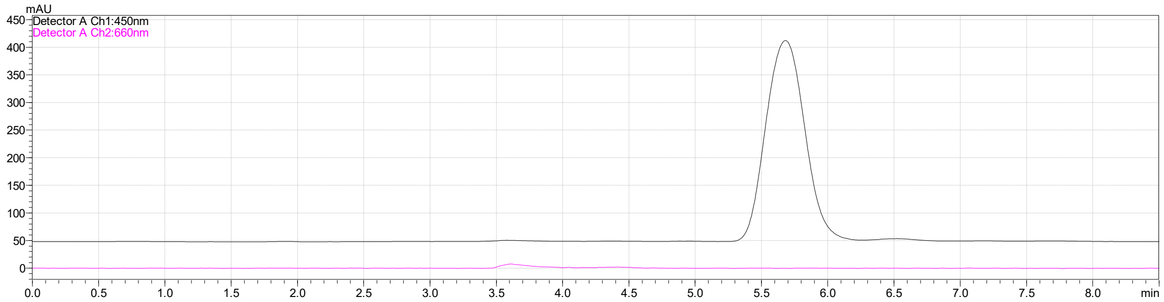

Figure S2. Chromatogram of the semi-preparative scale subfraction, analyzed in 450 nm—absorption region of fucoxanthin at a higher intensity (Au 450), Retention Time (RT) = 5.75 min black line b) 650 nm—region of the band's absorption of chlorophyll "a" lower intensity. RT ~ 3 min, pink line.

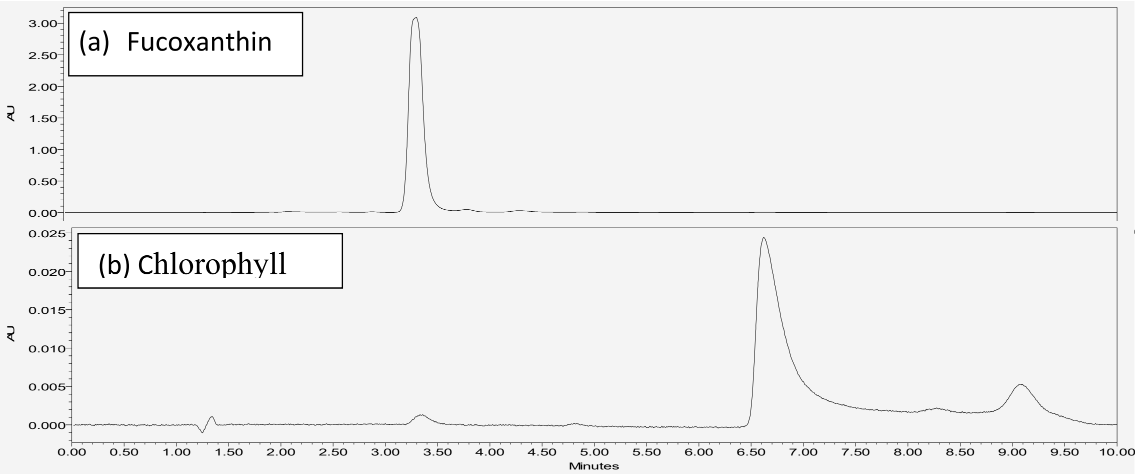

Figure S3. Chromatogram of the elution of the subfraction analytical scale, analyzed in (a) the 450 nm—absorption region of fucoxanthin at a higher intensity (3 Au), Retention time (RT) = 3.5 min; (b) 650 nm—the area of the chlorophyll absorption bands at a lower intensity (0.025 Au), RT ~ 6.5 min.
